# Rhein Reduces Fat Weight in *db/db* Mouse and Prevents Diet-Induced Obesity in C57Bl/6 Mouse through the Inhibition of PPAR**γ** Signaling

**DOI:** 10.1155/2012/374936

**Published:** 2012-09-25

**Authors:** Yu Zhang, Shengjie Fan, Na Hu, Ming Gu, Chunxiao Chu, Yiming Li, Xiong Lu, Cheng Huang

**Affiliations:** ^1^School of Pharmacy, Shanghai University of Traditional Chinese Medicine, Shanghai 201203, China; ^2^Scientific Experimental Center, Shanghai University of Traditional Chinese Medicine, Shanghai 201203, China

## Abstract

*Rheum palmatum* has been used most frequently in the weight-reducing formulae in traditional Chinese medicine. However, the components of *Rheum palmatum* that play the antiobesity role are still uncertain. Here, we tested the weight-reducing effect of two major *Rheum palmatum* compounds on *db*/*db* mouse. We found that rhein (100 mg kg^−1^ day^−1^), but not emodin, reduced the fat weight in *db*/*db* mouse. Using diet-induced obese (DIO) C57BL/6 mice, we identified that rhein blocked high-fat diet-induced obesity, decreased fat mass and the size of white and brown adipocytes, and lowered serum cholesterol, LDL cholesterol, and fasting blood glucose levels in the mice. To elucidate the underlying mechanisms, we used reporter assay and gene expression analysis and found that rhein inhibited peroxisome proliferator-activated receptor **γ** (PPAR**γ**) transactivity and the expression of its target genes, suggesting that rhein may act as a PPAR**γ** antagonist. Our data indicate that rhein may be a promising choice for antiobesity therapy.

## 1. Introduction

Obesity is one of the most serious public health problems of the 21st century [1, 2]. It increases the risk of various fetal diseases, particularly coronary artery disease, type II diabetes, hypertension, dyslipidemia, and certain types of cancer [3–5]. At present, there is only one drug, orlistat, approved by the Food and Drug Administration (FDA) for long-term use in the treatment of obesity. Thus, it is urgent to develop a new therapy for the prevention and treatment of obesity [5].

The development of novel antiobesity drugs has been proven difficult because of side-effects and lower efficiency [6–9]. Traditional medicine has various herbs for weight-reducing practice and may be a potential source for novel antiobesity drugs. *Rheum palmatum* is used most frequently in the weight-reducing formulae of traditional Chinese medicine. Recently, rhein, one of the major components of *Rheum palmatum,* has been shown to be an inhibitor of 3T3-L1 adipocyte differentiation [10]. Moreover, rhein has been reported to have pharmacological and biochemical effects on the inhibition of liver fibrosis and insulin sensitizing [11–13] and prevent hepatic steatosis through LXR inhibition in a high-fat diet-induced obese mouse model [14]. However, whether rhein plays the antiobesity role *in vivo* is still uncertain and the underlying mechanisms also need to be elucidated.

In the present study, we compared the weight-reducing effect of two major compounds from *Rheum palmatum*. We found that rhein reduced fat weight in *db*/*db* mouse. We also showed that rhein blocked weight gain and prevented hyperlipidemia and insulin resistance in high-fat diet-induced obese mice. Reporter assay and gene expression analysis verified that rhein inhibited PPAR*γ* transactivity and might play a role in prevention and treatment of obesity and metabolic diseases.

## 2. Material and Methods

### 2.1. Chemicals

**Figure 1:**
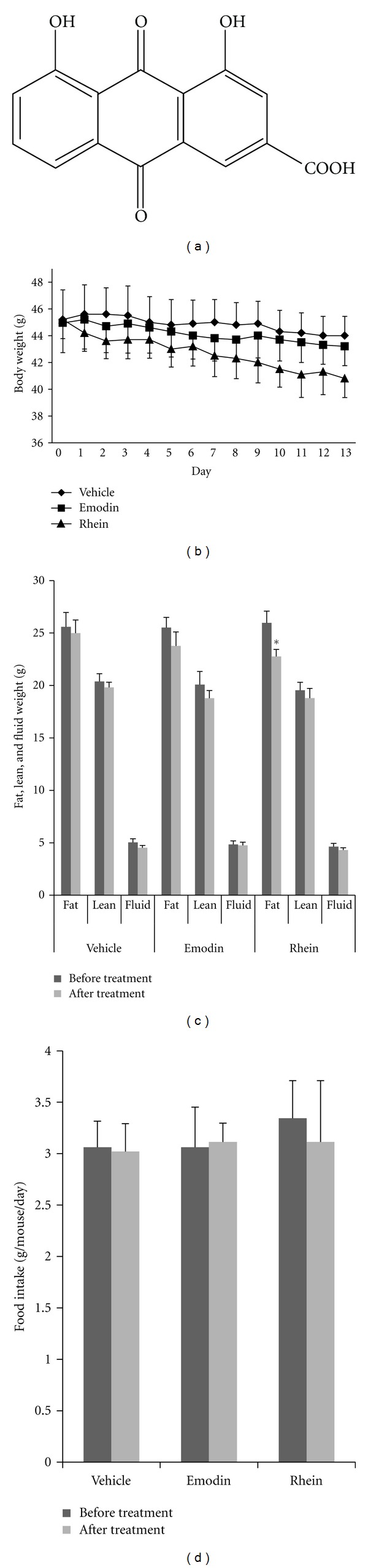
Effects of rhein on body and fat weight in *db*/*db* mice. (a) Structure of rhein. (b) Body weight. (c) Fat, lean, and fluid weight. (d) Food intake amount. The mice were dosed each day for 2 weeks with either emodin or rhein (100 mg kg^−1^ day^−1^) in a water vehicle or vehicle only using an oral gavage. The food intake amount was recorded every 24 hours through the treatment. Values are mean ± SE for 5 mice per group. *P < 0.05, versus vehicle control.

Rhein (Figure 1(a)) and emodin of 98% purity were purchased from the Nanjing Institute of Chinese Materia Medica (Nanjing, China) and were dissolved in dimethysulfoxide (DMSO). Rosiglitazone, GW0742, and WY-14643 were obtained from Sigma-Aldrich (St. Louis, MO, USA).

### 2.2. Animals

The animal protocols used in this study were approved by the Shanghai University of Traditional Chinese Medicine for animal studies (Approval number 10032). Female C57BL/6 mice and *db*/*db* mice (C57BL BKS cg-M^+/+^ lepr^−/−^) were purchased from the SLAC Laboratory (Shanghai, China). For induction of obesity, C57BL/6 mice with corresponding age and body weight were randomly separated into different groups and then placed on a high-fat (HF) diet (60% of calories derived from fat, Research Diets, New Brunswick, NJ; D12492), or the HF diet was composed of 0.1% rhein, while the low-calorie diet was the equivalent of a chow diet control (10% of calories derived from fat, Research Diets; D12450B). The diet research was started at 6 weeks of age and lasted for 8 weeks. Twenty-four-hour food intake was recorded in both treated groups and controls during the treatment. The experimental diets did not lead to any change in the daily food intake compared to control.

Eleven-week-old *db*/*db* mice were used in the experiment. The mice were dosed each day with either emodin or rhein (100 mg kg^−1^ day^−1^) using oral gavage in a water vehicle for 2 weeks. Control mice were given water with oral gavage. The weight of fat, lean, and fluid was measured with the Minispec TD-NMR system (Brucker, Germany).

### 2.3. Serum Chemistry Analysis

Serum triglyceride (TG), total cholesterol (TC), HDL cholesterol (HDL-c), and LDL cholesterol (LDL-c) were examined using a Hitachi 7020 Automatic Analyzer (Hitachi, Tokyo, Japan) with 100 *μ*L heart blood serum.

### 2.4. Scanning Electron Microscope

The adipose tissue was fixed in 10% neutral formalin and then fixed in 1% osmium tetraoxide. A Philip XL-30 scanning electron microscopy was used to examine the structure of fat tissue according to the previous described [9]. The diameter of the adipocytes was assayed by the method developed by Sugihara et al. [15]. 

### 2.5. Intraperitoneal Glucose Tolerance Test

C57BL/6 mice were fasted overnight (12 h) after 8 weeks of treatment. Glucose levels were determined from the tail vein (0 min) before the injection of glucose (1 g/kg body weight). Additional blood samples were collected at regular intervals (15, 30, 60, and 90 min) for glucose measurement. 

### 2.6. Rectal Temperature Measurement

The rectal temperature of mice was examined with a rectal probe attached to a digital thermometer (Physitemp, NJ, USA). At different times, body temperature of the mice was measured by a rectal probe after cold exposure as indicated (4°C).

### 2.7. Quantitative Real-Time PCR

According to the manufacturer's instructions, total RNA was extracted with a spin column (Qiagen, Germany) and reverse transcription into cDNA (Fermentas, Madison, WI, USA) was performed. Gene expression levels were measured by quantitative real-time RT-PCR conducted using the ABI StepOne Plus Real Time PCR system (Applied Biosystems, USA) according to the manufacturer's instructions. The primers involved in the experiments are displayed in Table 1. The cDNA was denatured at 95°C for 10 min followed by 40 cycles of PCR (95°C, 15 s, 60°C, 60 s). All results were obtained from at least three independent experiments. The experiment was normalized using beta-actin as internal control.

### 2.8. Transfection of Cultured Cells and Reporter Assays

The reporter assay was conducted as previously described [16, 17]. The expression plasmid pCMX-Gal-mPPAR*α*, *γ*, *β*/*δ*-LBD, and the Gal4 reporter vector MH100 × 4-TK-Luc were cotransfected. All transfections contained 10 *μ*g of total plasmids and 15 *μ*L FuGENE HD (Roche, Germany) per mL of DMEM. Transfection mixture was added to HEK293T cells for 24 hours, and the solution was changed to fresh media containing PPAR*γ*, PPAR*α*, and PPAR*β*/*δ* agonist rosiglitazone, WY14643 and GW0742 or rhein. Cells were then collected to determine luciferase activity 24 h later according to the protocol of the Dual-Luciferase Reporter Assay System (Promega, USA). The transfection efficiencies were normalized using renilla luciferase activity as an internal control. All the transfection experiments were performed in triplicate and repeated at least three times independently.

### 2.9. Statistical Analysis

The results are shown as mean ± SE. Data analyses were performed using SPSS12.0 for Windows statistical program. Statistical analysis was programmed by one-way and two-way analysis of variance (ANOVA). Difference was regarded as significant when *P* < 0.05.

## 3. Results

### 3.1. Rhein Reduces Fat Weight in *db*/*db* Mice

To test whether the *Rheum palmatum* compounds affected adipocytes *in vivo*, we observed the weight-reducing effects of emodin and rhein, two major components from *Rheum palmatum,* on *db*/*db* mice.  Figure 1(b) showed that rhein reduced the body weight of *db*/*db* mice (from 45.2 ± 1.4 g to 40.8 ± 1.4 g), whereas emodin did not display weight-reducing effects on *db*/*db* mice when compared to vehicle control after 2 weeks of treatment. We further measured the weight of fat, lean and fluid in *db*/*db* mice. As shown in Figure 1(c), rhein treatment significantly decreased the total fat weight (*P* < 0.05), but emodin did not. The lean and fluid weights remained unaffected between all groups. During the treatment, the food intake amount was no notable change in all groups (Figure 1(d)). The data suggested that rhein may decrease the fat mass *in vivo* and be responsible for the weight-reducing effect of *Rheum palmatum*. 

### 3.2. Rhein Blocks Weight Gain and Increases Energy Expenditure in DIO Mice

**Figure 2:**
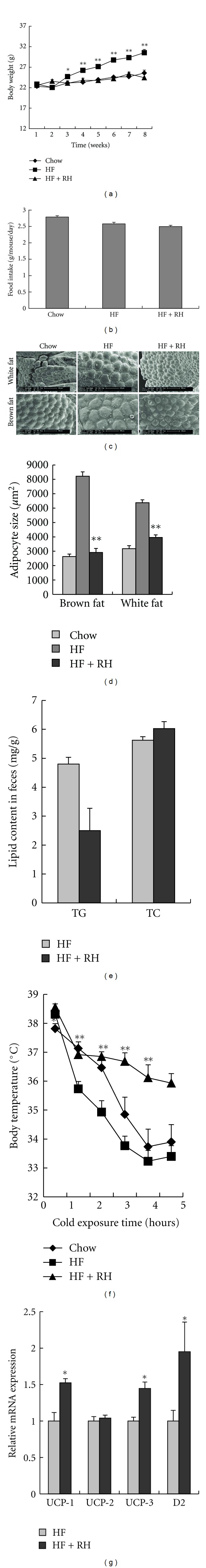
Rhein blocks body weight gain in high-fat diet-fed C57BL/6 mice. (a) Body weight. (b) Food intake amount. (c) The mass of white adipose tissue (WAT) and brown adipose tissue (BAT). (d) The adipocyte size of BAT and WAT in *μ*m^2^. (e) TG and TC content in feces. (f) Body temperature at different times after cold exposure (4°C). (g) The mRNA expression level of the thermogenic genes in BAT. *β*-actin was used as an internal control for modifying the mRNA level. Mice were fed a high-fat diet for 8 weeks and rhein was powdered and mixed in the diet at 0.1% (wt/wt). HF: high-fat diet. RH: rhein. The food intake amount was recorded every 2 days through the treatment. Values are mean ± SE for 7 mice per group. *P < 0.05, **P < 0.01 versus HF group.

To investigate whether rhein also blocks adipocyte formation *in vivo*, we assayed the effect of rhein on body weight gain induced by a high-fat diet in C57BL/6 mice. The C57BL/6 mice were fed HF diet alone or HF diet mixed with rhein (0.1%) for 8 weeks. At the end of treatment, the body weight of rhein-treated mice was significantly lower than that of HF control mice (Figure 2(a)), indicating that rhein could block body weight gain induced by the HF diet. During the feeding period, rhein-treated mice had a similar amount of food intake as that of control mice (Figure 2(b)), suggesting that the body weight reduction in the rhein-treated group may be not due to less calorie intake. Next, we examined the mass of adipocytes with scanning electron microscope. The results showed that rhein treatment reduced the cell size of both white adipocyte tissue (WAT) and brown adipocyte tissue (BAT), when compared to HF control mice (Figures 2(c) and 2(d)), indicating that rhein could protect against HF-induced increase in fat mass.

Since *Rheum palmatum* is a laxative agent, we determined the triglyceride and total cholesterol contents in mice feces to assay whether rhein inhibits lipid absorption in the intestine. The results showed that rhein treatment did not increase TG and TC contents in feces (Figure 2(e)), suggesting that the inhibition of obesity by rhein is not caused by inhibition of lipid absorption.

To see whether rhein increases energy expenditure, we measured body temperature, which is closely related to energy expenditure. Rhein-treated mice showed a markedly higher body temperature when exposed to cold (4°C) than HF control mice (Figure 2(f)). The increase in body temperature may contribute to the rise in energy expenditure, which prevents the development of obesity. To confirm this, real-time RT-PCR was performed to assay the expression level of thermogenic genes, UCPs and D2. The results showed that the expression of UCP-1, UCP-3, and D2 was markedly increased in BAT of the rhein-treated mice (Figure 2(g)). These data support that rhein may block body weight gain in DIO mice through enhancing energy expenditure.

### 3.3. Rhein Ameliorates the Lipid Profile and Glucose Tolerance in DIO Mice

**Figure 3:**
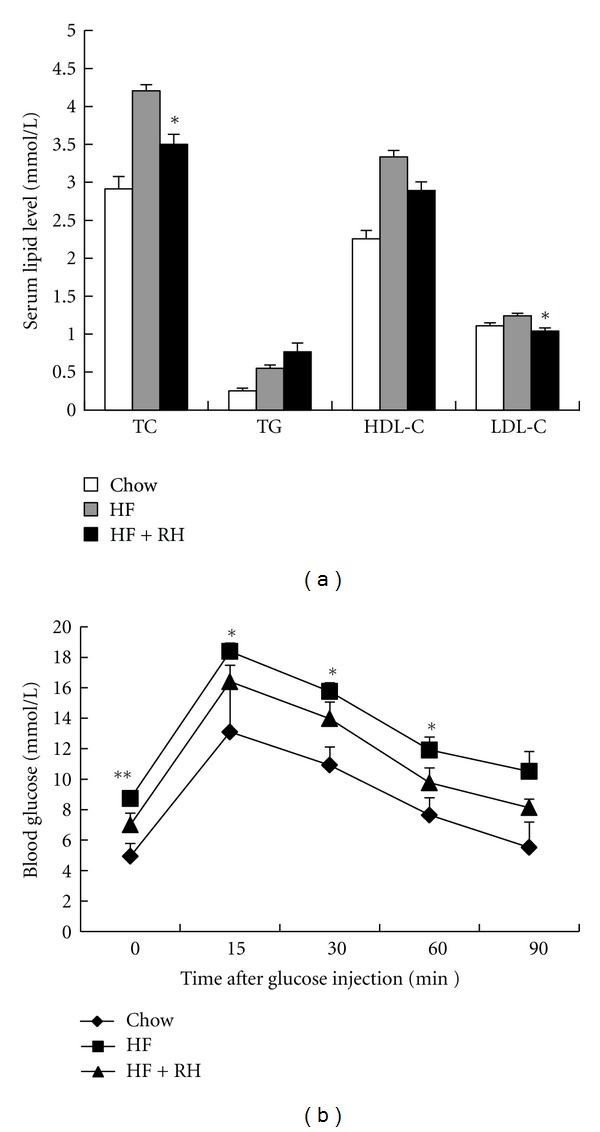
Rhein improves lipid profiles and glucose tolerance test in high-fat diet-induced obese C57BL/6 mice. (a) TC, TG, HDH-c, and LDL-c contents. (b) Fasting blood glucose levels (0 min) and glucose tolerance test (GTT). The mice were fasted for 12 hours before measuring blood glucose levels as 0 min. Then 1 g/kg body weight of glucose was injected intraperitoneally and glucose levels were tested at regular intervals of 15, 30, 60, and 90 min. Data are presented as mean ± SE for 7 mice per group. HF: high-fat diet. RH: rhein. *P < 0.05, **P < 0.01 versus HF group.

To understand lipid contents in blood, serum lipid levels were examined and were shown in Figure 3(a). Serum TC and LDL-c levels in rhein-treated C57BL/6 mice were significantly lowered when compared to that in HF fed mice. However, TG and HDL-c levels were not changed significantly.

It is well known that type 2 diabetes commonly coexists with obesity. The main basis for this connection is the ability of obesity to engender impaired glucose tolerance and insulin resistance [18–20]. To make sure that rhein improved insulin resistance *in vivo*, we measured fasting blood glucose levels and glucose tolerance in rhein-treated mice. The HF-fed mice exhibited higher fasting blood glucose levels and impaired glucose tolerance when compared to chow control mice (Figure 3(b)), while the rhein-treated group showed lower fasting glucose levels than HF-fed mice. Glucose tolerance in rhein-treated mice was also improved at 15, 30, and 60 min, indicating that rhein could ameliorate glucose tolerance and may prevent against the development of insulin resistance (Figure 3(b)). Collectively, these data demonstrate that rhein could improve metabolic disorders in diet-induced obese mice.

### 3.4. Rhein Inhibits the Transactivities of PPAR*γ*

**Figure 4:**
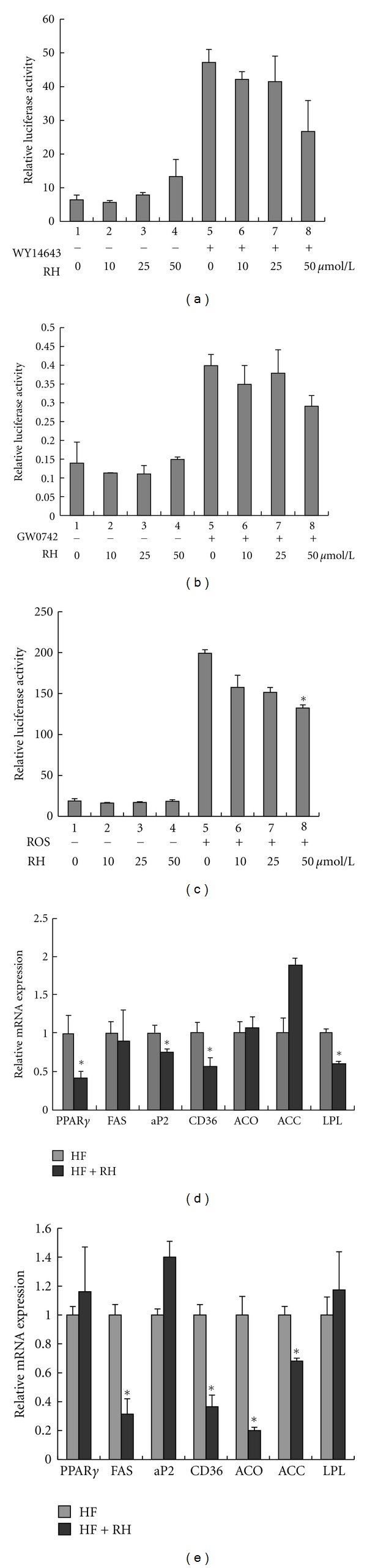
Rhein regulates the transcription activity of PPAR*γ*. ((a)–(c)) The transcription activity of PPAR*α*, *γ*, *β*/*δ*. GAL4-DBD-LBD expression plasmids and a GAL4-responsive luciferase reporter were cotransfected into HEK293T cells for 24 h and treated with the PPAR*γ*, PPAR*α*, and PPAR*β*/*δ* agonist rosiglitazone, WY14643, GW0742 (10 *μ*M), and rhein (10, 25, and 50 *μ*M) for another 24 h. Rellina-luc was used as the transfection efficiency control, and the relative luciferase activities were measured by comparison to renilla luciferase activities. The results represent at least three independent experiments, and data are presented as means ± SE. (d) The relative expression levels of PPAR*γ* target genes in WAT. (e) The relative expression levels of PPAR*γ* target genes in the liver tissue. *β*-actin was used as an internal control for modifying the mRNA level. HF: high-fat diet. RH: rhein. Values are mean ± SE for 7 mice per group. *P < 0.05 versus HF group.

Based on the inhibition of obesity and hyperlipidemia by rhein, we tested whether rhein affected PPARs, which are drug targets for metabolic syndromes. We transfected the GAL4-ligand-binding domain (LBD) of the nuclear receptor transcription factor fusion plasmids and a plasmid of the UAS reporter into HEK293T cells. There was no remarkable change in PPAR*α* and PPAR*β*/*δ* transactivities (Figures 4(a) and 4(b)). However, we found that rhein decreased the transactivity of PPAR*γ* activated by its agonist, rosiglitazone (Figure 4(c)), demonstrating that rhein may be an antagonist of PPAR*γ*.

Activation of PPAR*γ* elicits the expression of a variety of genes, including cluster of differentiation 36 (CD36), fatty acid synthase (FAS), adipose fatty acid-binding protein (aP2), acyl-CoA oxidase (ACO), acetyl coenzyme A carboxylase (ACC), and lipoprotein lipase (LPL) which are associated with adipogenesis, energy expenditure, and glucose metabolism. We analyzed the effects of rhein on the expression of PPAR*γ* target genes by investigating their mRNA expression levels in WAT and liver tissue of the mice. The results showed that rhein significantly inhibited the mRNA expression of aP2, CD36, LPL, and PPAR*γ* itself in WAT (Figure 4(d)). In liver tissue, rhein markedly decreased the expression of FAS, ACC, ACO, and CD36 (Figure 4(e)). Collectively, the data indicate that rhein plays a role in metabolic disorders by regulating PPAR*γ* signaling.

## 4. Discussion

*Rheum palmatum *is the most frequently used herb in weight-reducing therapy in traditional Chinese medicine. Here, we tested rhein, one major compound of *Rheum palmatum*, for its weight-reducing effects in obese mice. We found that rhein induced fat loss in *db*/*db* mice. Also, rhein blocked body weight gain in C57BL/6 mice induced by a high-fat diet through the inhibition of PPAR*γ* signaling. These results revealed that rhein is an active antiobesity component of *Rheum palmatum*.

The current antiobesity drugs on the market can be divided into two types: appetite inhibitors and lipid absorption inhibitors [21–26]. Similar to *ob*/*ob* mice, the *db*/*db* mouse model is useful for obesity studies. Rhein treatment significantly reduced fat weight in the *db*/*db* mice. We noticed that the food intake amount in rhein-treated mice was not decreased, which was also observed in the high-fat diet-feeding C57BL/6 mice, suggesting that the weight-reducing effect of rhein is not caused by suppressing appetite. 

*Rheum palmatum* is a laxative herb that causes diarrhea in patients, which may be similar to the weight-reducing drug orlistat, a pancreatic lipase inhibitor [27]. However, rhein did not result in diarrhea in both *db*/*db* and DIO mice. Lipid analysis showed that fecal TG content in rhein-treated mice was even lower than that in control mice, indicating that rhein is not an inhibitor of lipid absorption in the intestine. 

The increase of energy expenditure could protect against weight gain and obesity and is considered as a potential therapy for obesity. In the present study, we found that the body temperature of rhein-treated mice was higher than that of control mice when exposed to a cold condition. The expression of the thermogenic genes, UCP-1, UCP-3 and D2, was notably increased in rhein-treated mice. These data indicated that rhein may increase energy expenditure through the induction of the thermogenic genes. 

PPAR*γ* is a ligand-activated nuclear transcription factor that plays key roles in regulating glucose homeostasis, lipogenesis, and adipocyte differentiation [28, 29]. PPAR*γ* agonists have been proven to be potent insulin sensitizing agents for treating type II diabetes, but induce body weight gain in patients [30, 31]. Conversely, suppression of PPAR*γ* signaling by its antagonist may inhibit adipocyte differentiation, reduce body weight, and improve metabolic disorders in C57BL/6 mice [16, 17], suggesting that PPAR*γ* is a potential target for obesity therapy. Our study showed that rhein suppressed PPAR*γ* transactivity in the presence of the PPAR*γ* agonist, rosiglitazone, indicating that rhein may be a PPAR*γ* antagonist. PPAR*γ* regulates the expression of a group of genes, such as CD36, ACC, ACO, FAS, LPL, and aP2, which are related to fatty acid synthesis, oxidation, and adipogenesis. The expression of FAS, ACC, ACO, and CD36 was significantly inhibited in the liver tissue, while the expression of aP2, CD36, and LPL was markedly decreased in WAT of rhein-treated mouse. These results further support that rhein may reduce fat weight through the regulation of PPAR*γ* signaling.

In conclusion, we found that rhein reduces fat weight in *db*/*db* mice and prevents the development of obesity by a high-fat diet and ameliorates hyperlipidemia and glucose tolerance in C57BL/6 mice. Reporter assay and real-time PCR showed that rhein inhibits PPAR*γ* signaling. Our data suggest that rhein improves metabolic disorders through the PPAR*γ* antagonism and rhein may be a potential candidate for obesity therapy. 

## Figures and Tables

**Table 1:** Sequences of the primers used in real-time PCR.

Gene	Forward primer	Reverse primer
*β*-Actin	TGTCCACCTTCCAGCAGATGT	AGCTCAGTAACAGTCCGCCTAGA
FAS	CTGAGATCCCAGCACTTCTTGA	GCCTCCGAAGCCAAATGAG
LPL	ATCGGAGAACTGCTCATGATGA	CGGATCCTCTCGATGACGAA
PGC-1*β*	GGGTGCGCCTCCAAGTG	TCTACAGACAGAAGATGTTATGTGAACAC
aP2	CATGGCCAAGCCCAACAT	CGCCCAGTTTGAAGGAAATC
ACC	GAATCTCCTGGTGACAATGCTTATT	GGTCTTGCTGAGTTGGGTTAGCT
ACO	CAGCACTGGTCTCCGTCATG	CTCCGGACTACCATCCAAGATG
CD36	GCTTGCAACTGTCAGCACAT	GCCTTGCTGTAGCCAAGAAC
UCP-1	CATCACCACCCTGGCAAAA	AGCTGATTTGCCTCTGAATGC
UCP-2	GGGCACTGCAAGCATGTGTA	TCAGATTCCTGGGCAAGTCACT
UCP-3	TGGCCCAACATCACAAGAAA	TCCAGCAACTTCTCCTTGATGA
D2	ACACCGTCGTCCGCAAA	CCCACCCACTCTCTGACTTTCT
PPAR*γ*	CGCTGATGCACTGCCTATGA	AGAGGTCCACAGAGCTGATTCC

## Abbreviations

ACC: Acetyl coenzyme A carboxylase
ACO: Acyl-CoA oxidase
aP2: Adipose fatty acid-binding protein
BAT: Brown adipocyte tissue
CD 36: Cluster of differentiation 36
D2: Deiodinase 2
DIO: Diet-induced obese
FAS: Fatty acid synthase
HDL-c: HDL cholesterol
IPGTT: Intraperitoneal glucose tolerance test
LDL-c: LDL cholesterol
LPL: Lipoprotein lipase
PGC1-*β*: Peroxisome proliferator-activated receptor gamma coactivator 1-beta
PPAR*γ*: Peroxisome proliferator-activated receptor *γ*
TC: Total cholesterol
TG: Triglyceride
UCP: Uncoupling protein
WAT: White adipocyte tissue.
